# Rapid Authentication of *Ginkgo biloba* Herbal Products Using the Recombinase Polymerase Amplification Assay

**DOI:** 10.1038/s41598-018-26402-8

**Published:** 2018-05-22

**Authors:** Yang Liu, Xiao-yue Wang, Xue-min Wei, Zi-tong Gao, Jian-ping Han

**Affiliations:** Institute of Medicinal Plant Development, Chinese Academy of Medical Sciences & Peking Union Medical College, Beijing, 100193 China

## Abstract

Species adulteration in herbal products (HPs) exposes consumers to health risks. Chemical and morphological methods have their own deficiencies when dealing with the detection of species containing the same active compounds in HPs. In this study, we developed a rapid identification method using the recombinase polymerase amplification (RPA) assay to detect two species, *Ginkgo biloba* and *Sophora japonica* (as adulteration), in *Ginkgo biloba* HPs. Among 36 *Ginkgo biloba* HP samples, 34 were found to have *Ginkgo biloba* sequences, and 9 were found to have *Sophora japonica* sequences. During the authentication process, the RPA-LFS assay showed a higher specificity, sensitivity and efficiency than PCR-based methods. We initially applied the RPA-LSF technique to detect plant species in HPs, demonstrating that this assay can be developed into an efficient tool for the rapid on-site authentication of plant species in *Ginkgo biloba* HPs.

## Introduction

Herbal products (HPs) diversely refer to products prepared using plant-derived materials and include dietary supplements, teas, extracts, natural health products and traditional medicines^1^. HPs are widely used to treat a variety of health conditions and highly demanded because of their multiple nutritional and medical values^2^. The World Health Organization (WHO) reported that traditional herbal medicines contribute $83 billion US dollars to the global HP market, and international HP trade shares have rapidly increased at approximately 15% annually^3,4^. However, the lack of standardized methods for the quality assessment of HPs poses serious risks to consumers worldwide. Misidentification and substitution of raw materials are the main factors that cause safety problems. Raw materials, such as bark, roots, leaves and seeds, from different plant species must be processed into capsules, tablets, teas or powders during the production process; thus, using a morphological method (even under a microscope) to identify processed herbals in these products is not suitable. Chemical methods are required for the quality assessment of HPs to test the main active components according to the Chinese Pharmacopeia (2015); however, chemical methods are also not sufficient for the assessment of HPs because they are complex botanicals and not single-active ingredient products. Chemical methods always encounter barriers when distinguishing different herbal species that share similar chemical compounds within the complex prescription^5^. Species adulteration and substitutions with chemical equivalents have had seriously adverse consequences on the health and safety of consumers^6^.

*Ginkgo biloba* extract, one of the most popular and scientifically explored HPs, is sold worldwide for its symptomatic treatment of deficiencies in memory, concentration, and depression due to organic brain diseases^7^. Flavone glycosides (24%) are the standard testing components of commercially sold *Ginkgo biloba* HPs^8^, and their content in *Sophora japonica* extract is also quite high. *Sophora japonica* is derived from a completely different genus and has low costs. Therefore, raw *Sophora japonica* materials can be deliberately adulterated as fillers in *Ginkgo biloba* extract to meet market levels and cut production costs. Chemical studies have made only an assumption about this adulteration according to the chemical profile of the detected HP samples^9,10^. A DNA-based molecular method is still required to confirm both the listed species ingredients and unlisted fillers used in HPs.

An on-site detection method using a DNA-based technique will help solve the adulteration problem in HPs. In recent years, researchers have paid increasing attention to DNA-based methods for the detection of HPs^4,11,12^. Polymerase chain reaction (PCR) is considered a gold standard detection protocol for most DNA-based methods. However, PCR requires high-cost equipment, such as electrophoresis systems, sensors and fluorescence detection optics^13^, and detecting PCR amplification results is commonly labour-intensive and has costly read-out processes. These requirements definitely hinder PCR-based methods from being developed into a rapid on-site authentication method for non-scientist users, such as enterprises and government regulators^14^. Thus, development of an efficient technique applicable to the on-site detection of HPs is necessary.

Recombinase polymerase amplification (RPA) is a recently developed isothermal amplification method that offers high sensitivity and specificity for DNA detection^15^. RPA can complete an entire reaction at a constant and low temperature, lower than 40 °C^16^. This technique includes a nucleoprotein complex formed by the recombinase enzyme, a oligonucleotide primer and DNA polymerase I^17^. The nucleoprotein complex and oligonucleotide primer facilitate strand exchange, and DNA polymerase I, a *Staphylococcus aureus* homologue, elongates the primer. RPA is more rapid, convenient and efficient than traditional PCR amplification^18^. For non-specialists and detection conditions with time and resource constraints, less complicated devices, such as lateral flow strips (LFS), can be preferentially selected to detect amplification results^19^. RPA-based testing combined with the LFS assay has been successfully utilized in the fields of *in vitro* diagnostics, virus detection and pathogen authentication^20–22^. However, no use of this assay for the rapid on-site identification of HPs has been developed or reported.

Herein, we aimed to develop the RPA-LFS assay as a rapid on-site detection method to identify *Ginkgo biloba* and *Sophora japonica* (as adulterants) ingredients in *Ginkgo biloba* HPs.

## Results

### Development of species-specific sequences, primers and probes

A 114-bp sequence unique to *Ginkgo biloba* was generated from the alignment of 27 chloroplast rbcL *Ginkgo biloba* sequences, and a 104-bp sequence unique to *Sophora japonica* was generated from the alignment of 24 internal transcribed spacer 2 (ITS2) *Sophora japonica* sequences. Details regarding these two sequences are provided in the Supplementary Information. Basic Local Alignment Search Tool (BLAST) results showed that only the targeted species have the corresponding unique sequences found in this study (Supplementary Figure S1). The species specificities of these two sequences were therefore ensured, and the presence of the species was confirmable by the presence of these two sequences in DNA extracted from HP samples. The species specificities of the probes were also evaluated by BLAST, showing 100% similarity with the sequences of the targeted species in the NCBI database except for two *Sophora japonica* sequences, which had similarities as high as 98% (Supplementary Figure S2). Details on the primers and probes designed in this study are listed in Table 1.

**Table 1:** Details regarding the primers and probes used in this study.

Primers used for PCR amplification				
Name	Direction	Amplicon Size	Targeted Species	Sequence (5′-3′)
YXF	Forward	114	*Ginkgo biloba*	CGAGGAAGGTTCTGTTGCTA
YXR	Reverse	114	*Ginkgo biloba*	TTGGAATAAGCAGGAGGAAT
HMF	Forward	101	*Sophora japonica*	GAGTCTTTGAACGCAAGTTG
HMR	Reverse	101	*Sophora japonica*	GACGGCACGGATGCTTAA
**Primers and probes used for RPA amplification**				
YXSF	Forward	176	*Ginkgo biloba*	ATTCCTCCTGCTTATTCCAAAACTTGCCAGGGTCC
YXSR	Reverse	176	*Ginkgo biloba*	biotin-TCAAGTCCACCACGAAGACATTCGTAAACTGCT
YX-probe	Forward	/	*Ginkgo biloba*	FAM-AATATGGCCGTCCCCTATTGGGATGTACTATC-THF-AGCC AAAATTGGGTT-C3-spacer
HMSF	Forward	132	*Sophora japonica*	TAGGTCCTGAGCGGGGCGAATGTTGGCTTC
HMSR	Reverse	132	*Sophora japonica*	biotin-AGACACACGCGATTGGTCTCGAGATTTTACTCAGC
HM-probe	Forward	/	*Sophora japonica*	FAM-CTTCCCGTGAGCCTTGTCTCGCGGTTGGTT-dspacer-AAAAA TGTGTCTGTGG-C3-spacer

### Validity of species-specific sequences, primers and probes

Pure DNA and a DNA mixture of these two species were amplified using the PCR and RPA techniques to verify the utilities of the species-specific sequences, primers and probes. Species-specific motifs were successfully sequenced after PCR amplification with pure DNA and the DNA mixture using the primers YXF/YXR and HMF/HMR, as expected. Positive RPA results were obtained by LFS after amplification with pure DNA and the DNA mixture using manually designed primers, YXSF/YXSR and HMSF/HMSR, and probes. The validity result was shown as positive control in subsequent experiments.

### Detection of HP samples with PCR-based methods and sequencing

**Figure 1:**
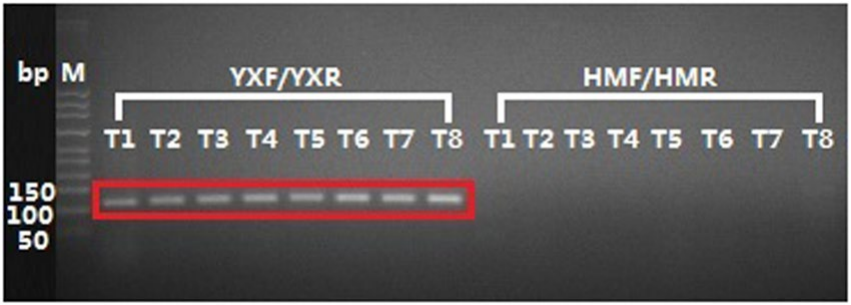
PCR Amplification of Tea with Primer Pairs YXF/YXR and HMF/HMR. Lanes 1 through 8 display the PCR results of amplifying tea samples with the primer pair YXF/YXR. Lanes 9 through 16 display the PCR results of amplifying tea samples with the primer pair HMF/HMR. The last lane displays the negative control. PCR products of all 8 tea samples were obtained with the primer pair YXF/YXR. No PCR products were obtained with the primer pair HMF/HMR.

**Figure 2:**
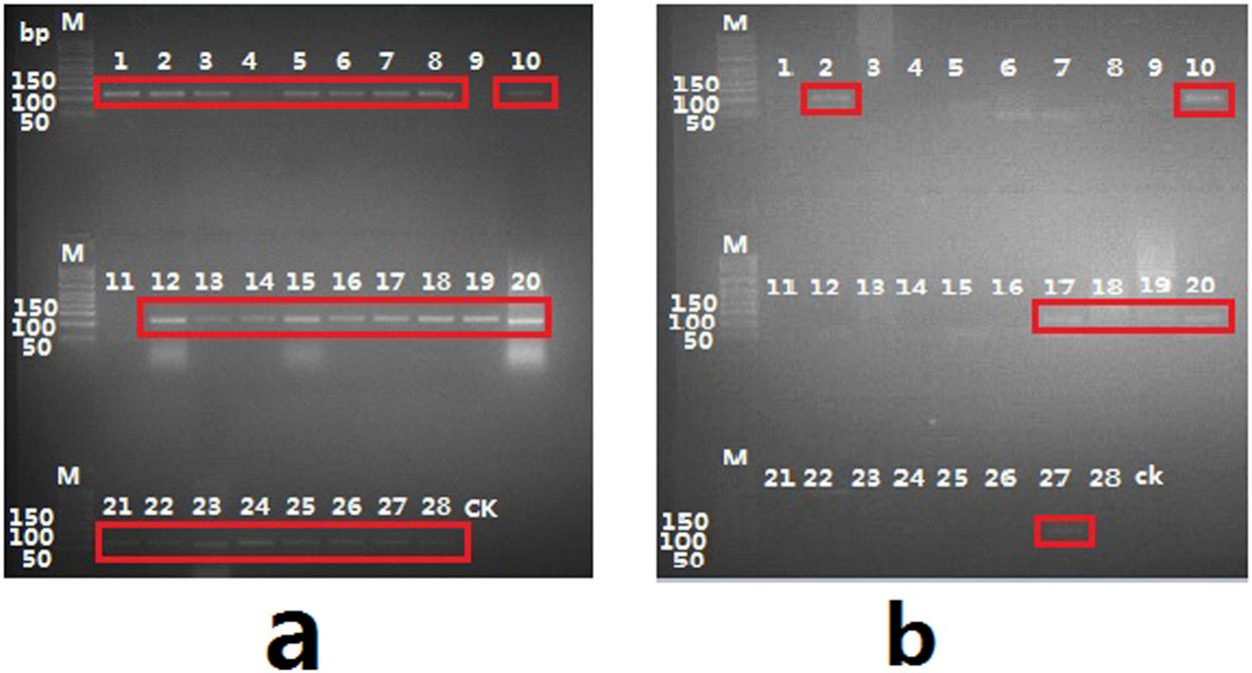
PCR Amplification of NHP with the Primer Pairs YXF/YXR and HMF/HMR. (**a**,**b**) Are a combination of two different gels with different exposures. (**a**) Shows PCR products amplified with the primer pair YXF/YXR. The last lane displays the negative control. (**b**) Shows PCR products amplified with the primer pair HMF/HMR. (**b**) Shows the overexposure modification to enhance the band luminosities. The last lane displays the negative control. The lanes in red frames show successfully sequenced PCR products.

To determine whether the HP samples contained *Sophora japonica* or *Ginkgo biloba*, DNAs extracted from tea and natural health products (NHPs) were amplified by PCR using the primers YXF/YXR and HMF/HMR. The PCR products were visualized after gel electrophoresis, and the bands in the red frames were targeted sequences that were successfully sequenced (Figs 1 and 2). *Ginkgo biloba* ingredients were found in all 8 tea samples, and no *Sophora japonica* ingredients were observed (Fig. 1). Except for those of samples NHP09 and NHP11, the PCR products of all other NHP samples were successfully sequenced and identified to contain *Ginkgo biloba* (Fig. 2a). The gel shown in Fig. 2b was overexposed to enhance the luminosity of the bands. The effect comparison of this gel under multiple exposures is shown in Supplementary Figure S3. Although the luminosities of bands in lanes 2, 10, 17, 18, 19, 20 and 27 were hardly visible even when overexposed (Fig. 2b), the PCR products concentrated with the primer pair HMF/HMR in the corresponding lanes were still successfully sequenced. PCR products of the other HP samples amplified with the primer pair HMF/HMR were neither visible nor sequenced. The sequencing results of all HP samples are summarized in Supplementary Table S1.

### Detection of HP samples with the RPA-LFS assay

**Figure 3:**
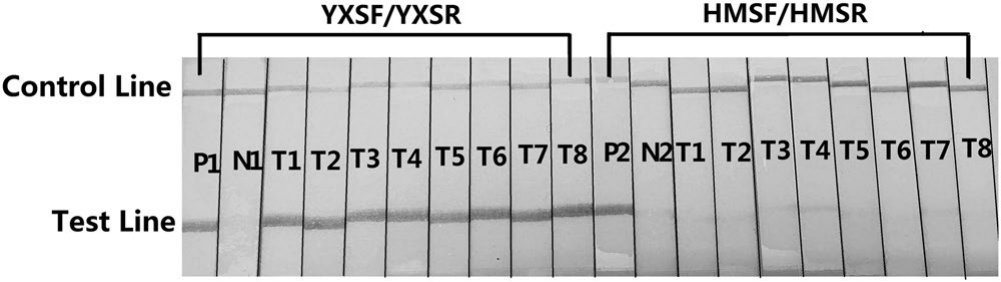
RPA-LFS Detection of Tea Samples. P1 represents the positive control for the *Ginkgo biloba* RPA-LFS detection system: detection results of the RPA amplification of DNA extracted from *Ginkgo biloba* leaves; N1 represents the negative control for the *Ginkgo biloba* RPA-LFS detection system. T1 through T8 show the detection results of the amplification of tea samples with the *Ginkgo biloba* RPA-LFS detection system. P2 represents the positive control for the *Sophora japonica* RPA-LFS detection system: detection results of the RPA amplification of DNA extracted from *Sophora japonica* leaves; N2 represents the negative control for the *Sophora japonica* RPA-LFS detection system. T1 through T8 show the detection results of amplifying tea samples with the *Sophora japonica* RPA-LFS detection system.

**Figure 4:**
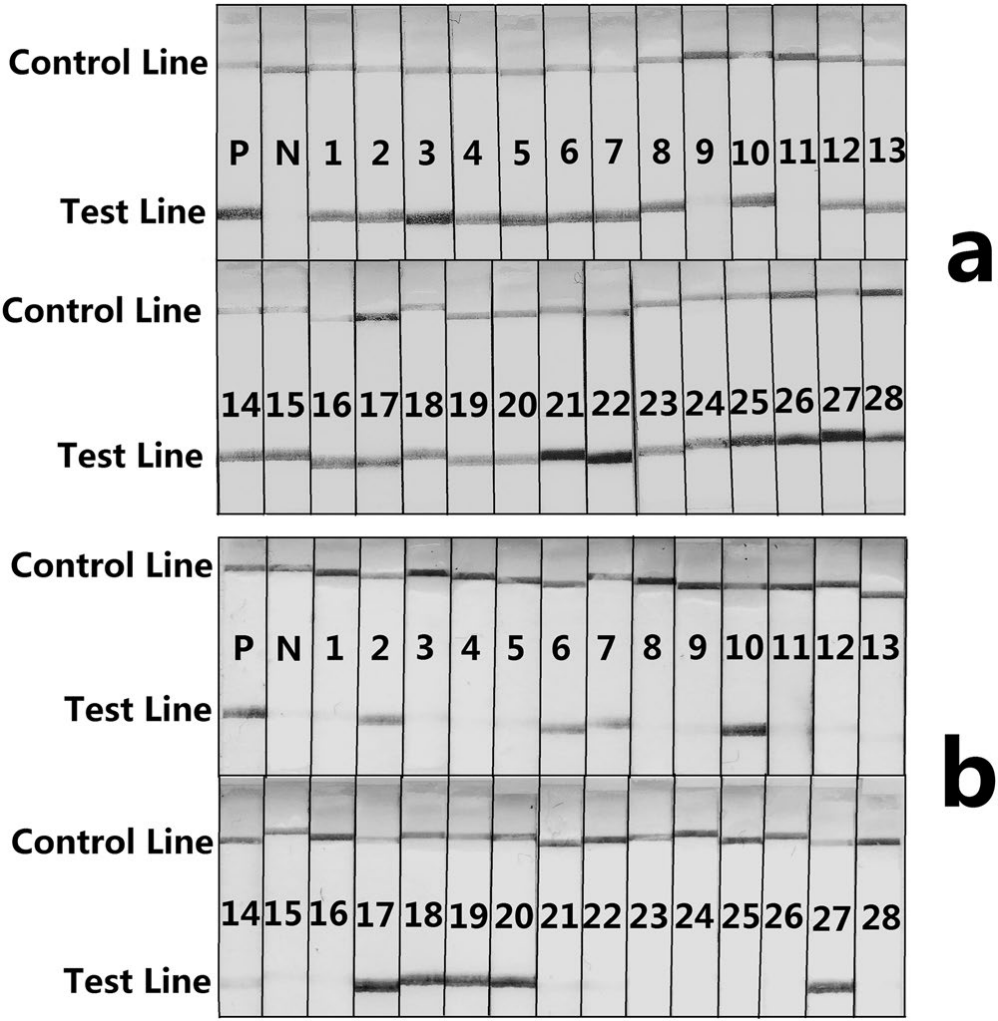
RPA-LFS Detection of *Ginkgo biloba* and *Sophora japonica* in NHP Samples. In (**a**) P represents the positive control for the *Ginkgo biloba* RPA-LFS detection system: detection results of the RPA amplification of DNA extracted from *Ginkgo biloba* leaves; N represents the negative control for the *Ginkgo biloba* RPA-LFS detection system. In (**b**) P represents the positive control for the *Sophora japonica* RPA-LFS detection system: detection results of the RPA amplification of DNA extracted from *Sophora japonica* leaves; N represents the negative control for the *Sophora japonica* RPA-LFS detection system.

To verify the utility of the RPA method, DNAs extracted from tea and NHPs were amplified with the primers YXSF/YXSR and HMSF/HMSR and the probes YP and HP. The detection results of 8 tea samples, visualized on LFS tests and obtained using the RPA-LSF assay, were in 100% accordance with those obtained using the PCR-based method and sequencing (Fig. 3). The RPA-LSF detection results of NHPs amplified with the primer pair YXSF/YXSR also perfectly matched the PCR results (Fig. 4a). However, in the RPA-LSF detection of NHPs amplified with the primer pair HMSF/HMSR, adulterated *Sophora japonica* ingredients were detected in 9 samples (Fig. 4b). Among these 9 samples, NHP06 and NHP07 were detected to have *Sophora japonica* ingredients that were not observed in the PCR-amplified products.

### Sensitivity and specificity of the RPA-LFS assay

**Figure 5:**
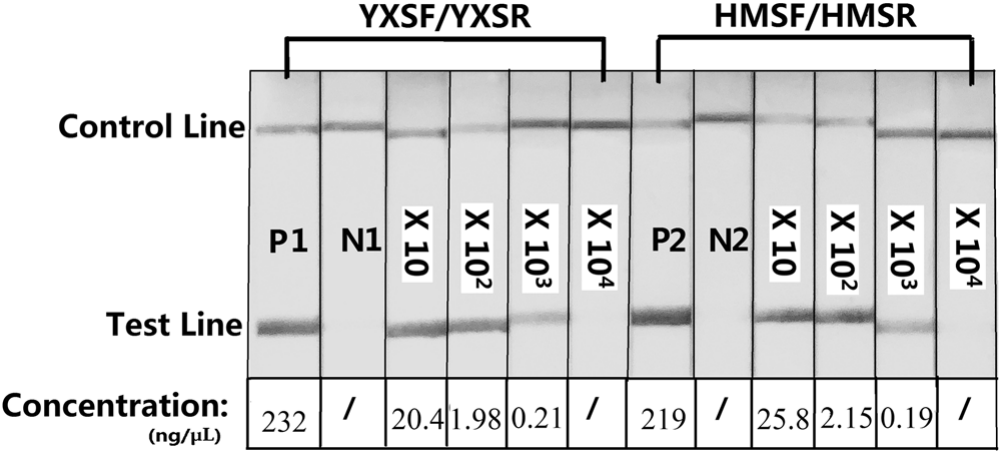
RPA-LFS Detection of Diluted DNA. P1 represents the positive control for the *Ginkgo biloba* RPA-LFS detection system: detection result of the RPA amplification of DNA extracted from *Ginkgo biloba* leaves; N1 represents the negative control for the *Ginkgo biloba* RPA-LFS detection system. The following strips showed the amplification of *Ginkgo biloba* DNA diluted as indicated on the label. P2 represents the positive control for the *Sophora japonica* RPA-LFS detection system: detection result of the RPA amplification of DNA extracted from *Sophora japonica* leaves; N2 represents the negative control for the *Sophora japonica* RPA-LFS detection system. The following strips showed the results of amplifying *Sophora japonica* DNA diluted as indicated on the label. The concentrations of diluted DNA are presented under the strips.

**Figure 6:**
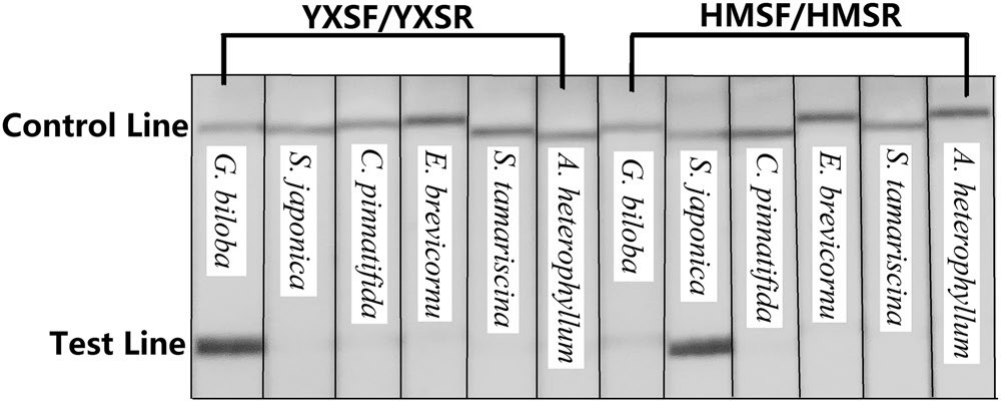
RPA-LFS Detection of DNA from Flavone-related Species. The species of tested DNA were labelled on the strips as indicated.

The RPA amplification products of DNA diluted 1 to 3 times were successfully detected by LFS, while the amplification of DNA 10-fold serially diluted 4 times was not detectable (Fig. 5). After diluting pure DNA 104 times, the concentration of the diluted DNA was too low to be measured. Approximately 1 ng of purified DNA was thus determined to be required for the RPA-LFS assay. The specificity test results for the RPA-LFS assay (Fig. 6) indicated that the assay was capable of detecting the targeted species ingredients with high specificity among other flavone-abundant species, including *Crataegus pinnatifida*, *Epimedium brevicornu*, *Selaginella tamariscina* and *Arisaema heterophyllum*.

## Discussion

Because the adulteration rate of commercial HPs is indeed high, identifying HPs at the molecular level is necessary. In our previous studies, we focused on the identification of ingredients in HPs based on species-specific sequences^13,23,24^. We used PCR electrophoresis and sequencing techniques in different cases. We detected high adulteration rates in various herbal products, including American ginseng, *Agelicae sinensis*, and *Lonicera japonica*. This study also indicated high adulteration rates in manufactured *Ginkgo biloba* products. In total, unlisted *Sophora japonica* ingredients were detected in 9 of 36 HPs. Among these HP samples, no adulteration was detected in tea samples, most likely because tea products are always obtained by simple processes, and some morphological characteristics of the species can thus be reserved. Compared to tea, products in powder, tablets or capsules are much more prone to adulteration due to their lack of traceable morphological and chemical characteristics. Together, these results demonstrate that more attention must be paid to the assessment and quality control of HPs. Molecular authentication methods have proven to be powerful tools for making this assessment and acting as quality control systems^25^.

Although DNA-based identification methods have substantial advantages for assessing HPs, obstacles remain that must be overcome^26^. First, raw herbal materials always undergo rigorous manufacturing procedures, such as boiling or grinding, which unavoidably lead to various degrees of DNA degradation. DNA degradation further decreases the rate of successfully PCR-amplifying molecular markers, such as traditional DNA barcode ITS and ITS2 sequences^27^. As a result, these molecular markers may fail to identify species ingredients in HPs. In contrast, shorter sequences are proposed to more efficiently identify HPs. Our previous studies proposed a new molecular marker method, termed the nucleotide signature, that utilizes a very short, species-specific sequence. The nucleotide signature method showed high identification success rates in herbal medicine products. In this study, our short, species-specific probes, primers and amplification targets were designed based on nucleotide signature sequences. Consequently, only two of 36 *Ginkgo biloba* HP samples failed to contain *Ginkgo biloba* sequences, most likely due to serious DNA degradation. Second, traditional PCR-based methods have the potential disadvantage of low accuracy in the downstream detection of amplification products. In this study, we used electrophoresis to first detect the PCR products and then utilized the sequencing technique to determine the presence of species-specific sequences in the HP samples. PCR products of *Sophora japonica*-specific sequences in samples NHP 18, NHP 19 and NHP 27 were hardly visible in an ethidium bromide-stained gel; however, they were successfully sequenced. Consequently, simply relying on the PCR electrophoresis detection system is not sufficiently accurate. By contrast, the RPA-LFS detection results conformed with the sequencing results, demonstrating that the accuracy of the RPA-LFS method is higher than that of the traditional PCR-based method. Third, some disadvantages of the sequencing technique procedure do exist, including long test periods, high costs and potential judgement inaccuracies caused by sequencing contamination^12,28^. In this study, we used the RPA-LSF assay to circumvent the sequencing process, thus avoiding these possible disadvantages.

The RPA-LSF assay has a series of unique advantages in many aspects. First, we herein showed that this assay has a high detection sensitivity. PCR products were not obtained from the NHP 06 and NHP 07 samples amplified with the primer pair HMF/HMR, while *Sophora japonica* DNA in these two samples was successfully amplified and detected by the RPA-LSF assay, demonstrating that the RPA-LSF assay indeed had a higher sensitivity than the PCR-based method. In addition, this conclusion was also directly drawn by comparing the sensitivity measurements in this study (1 ng) to those of our previous study (8 ng)^13^. Yang *et al*. reported that the sensitivity of RPA is lower than that of RT-qPCR^29^; however, its sensitivity can still be improved using self-avoiding molecular recognition systems (SAMRS)^30^. Second, the RPA-LSF assay has a high amplification efficiency of short sequences, especially of those no more than 200 bp in length. This specialty makes the RPA technique an ideal choice for circumventing potential DNA degradation in HPs. Additionally, the entire detection process after DNA extraction can be completed in only 30 min without complicated equipment or skills. This would provide supervision department officers higher work efficiency than any of the other methods discussed thus far. Finally, the RPA-LSF assay is highly specific for the detection of targeted species because of species-specific probes and specially designed primers. The specificity test showed that no cross-reactivity existed between *Ginkgo biloba* and *Sophora japonica*, and the assay detected only the targeted species among the other common plant species containing abundant flavone compounds. Due to its high specificity, the sequencing technique could be avoided to save time and cost. However, the extreme high species-specificity of this assay is sometimes a double-edged sword. On the one hand, accurate detection results are obtained, but on the other hand, the high species-specificity may not be convenient when multiple species in HPs must be detected. Fortunately, the RPA assay has been reported to simultaneously amplify and detect DNA from multiple parasites when used in combination with specially designed LFS assays^31^. Similar systems can be developed for the simultaneous rapid authentication of multiple species components in HPs.

## Conclusion

The RPA-LSF assay was initially developed to detect *Ginkgo biloba* and *Sophora japonica* in HPs. In this study, the high efficiency, specificity and sensitivity of this assay were highlighted. The RPA-LSF assay overcame the disadvantage of chemical methods in identifying different species with similar chemical profiles. This assay could replace PCR-based methods and be used as a rapid on-site identification method for HPs without requiring advanced equipment or complicated professional skills. Further development of this assay for other herbal species could greatly expand the application of commercial HP molecular authentication. Undoubtedly, the utility of the RPA-LSF assay in regulating HPs will help further complete assessment and quality control systems and significantly benefit science, industry and consumers.

## Methods and Materials

### Materials and species-specific sequences

A total of 36 *Ginkgo biloba* HP samples were bought online, including 28 samples of *Ginkgo biloba* leaf extract and 8 samples of *Ginkgo biloba* leaf tea. The samples of *Ginkgo biloba* leaf extract used in this study were produced in five different countries, America, Australia, China, Germany and Japan, and all the tea products were produced in China. Details regarding these samples are listed in Table S1. The manufacturers’ names are not shown to protect their identities. A total of 27 chloroplast rbcL *Ginkgo biloba* sequences were downloaded from the NCBI database, and 24 ITS2 *Sophora japonica* sequences were generated from our previous study. To search short and species-specific motifs, the sequences were aligned using MEGA software, and the BLAST method was used to verify the species specificities of the motifs.

### Primers pairs and probe design

Potential primers for PCR amplification of the species-specific motifs were designed using Primer 6.0 software. The primers and probes used for RPA were manually designed based on suggestions given by TwistDx instruction (www.twistdx.co.uk).

### Verification of species-specific sequences, primers and probes

To obtain a positive control, pure *Ginkgo biloba* and *Sophora japonica* DNA samples were extracted from the leaves of these two species using a Tiangen Plant DNA Kit (Tiangen Biotech, China) according to the manufacturer’s instructions.

*Ginkgo biloba* DNA was amplified with primers YXF/YXR in a 25 µL volume comprising 12.5 µL of 2× Taq PCR Mix (Tiangen Biotech), 2.0 µL of forward and reverse primers (2.5 µM, total of 4.0 µL of primers), 2.0 µL of template DNA, 1.0 µL of Mg2+ and 5.5 µL of sterile water. After optimization, PCR amplification of the *Ginkgo biloba*-specific sequence was performed under the following conditions: initial denaturation at 95 °C for 2 min, followed by 40 cycles of denaturation at 94 °C for 1 min, annealing at 45 °C for 30 s, extension at 72 °C for 30 s, and a final elongation step at 72 °C for 7 min. Similarly, after optimization, *Sophora japonica* DNA was amplified with primers HMF/HMR in a 25 µL volume under the following conditions: initial denaturation at 95 °C for 2 min, followed by 40 cycles of denaturation at 94 °C for 1 min, annealing at 52 °C for 30 s, extension at 72 °C for 30 s, and a final elongation step at 72 °C for 7 min. Then, the DNA mixtures of these two species were amplified with primers YXF/YXR and HMF/HMR. All the PCR reactions were carried out using the GeneAmp® PCR 9700 system (Applied Biosystems, Foster City, CA). The PCR products were bi-directionally sequenced by the Major Engineering Laboratory of the Chinese Academy of Agricultural Sciences University.

RPA amplifications of *Ginkgo biloba* DNA, *Sophora japonica* DNA and the DNA mixture were performed with primers YXSF/YXSR and HMSF/HMSR using the Twist Amp nfo kit (TwistDx, Cambridge, United Kingdom). RPA amplification was carried out in a 50 µL volume comprising 2.1 µL of forward and reverse primers (10 µM), 0.6 µL of probe (10 µM), 29.5 µL of rehydration buffer, 5 µL of template DNA, 8.2 µL of sterile water and 2.5 µL of MgAc. The mixture was incubated in a water bath at 39 °C for 20 min, and 5 µL of the amplification product was then diluted in 45 µL of PSBT assay buffer. Next, 10 µL of each diluted RPA product was loaded on the lateral flow dipstick (Milenia Biotec GmbH, Germany), which was inserted into 200 mL of PSBA running buffer.

### DNA extraction, amplification and sequencing to verify the species in HPs

For the extraction of DNA from *Ginkgo biloba* tea leaves, a total of 20–30 mg of tea was placed into a 2.0-mL centrifuge tube. Then, the sample was milled using a ball-milling machine (Restch, Germany), and genomic DNA was isolated from the resulting powders using a Tiangen Plant DNA Kit (Tiangen Biotech) according to the manufacturer’s instructions. Total DNA from one sample was finally dissolved in 50 µL of sterile water. For the extraction of DNA from *Ginkgo biloba* leaf extract, a total of 50–60 mg of extract was placed into a 2.0-mL centrifuge tube; six tubes were run in parallel for one sample. Three tubes were eluted through one absorption column, and two absorption column products from one sample were finally dissolved in 50 µL of sterile water. Except for the number of tubes ran in parallel, all protocols for the tea and extract samples were the same.

The species-specific sequences within DNA extracted from 36 HP samples were amplified by PCR with primers YXF/YXR and HMF/HMR using the same volumes and conditions described above. The PCR products were bi-directionally sequenced by the Major Engineering Laboratory of the Chinese Academy of Agricultural Sciences University.

### Recombinase polymerase amplification to verify the utility of the RPA-LFS assay

RPA amplification of DNA isolated from 36 HP samples was performed with primers YXSF/YXSR and HMSF/HMSR according to the manufacturer’s instructions (TwistDx, www.twistdx.co.uk). The amplification products were detected using a lateral flow dipstick (Milenia Biotec GmbH). Then, the detection results of the RPA-LFS assay were compared with the sequencing results of each sample.

### Determination of the sensitivity and specificity of the RPA-LFS assay

To determine the threshold amount of DNA needed for the RPA-LSF assay, pure *Ginkgo biloba* and *Sophora japonica* DNA extracted from raw leaves was ten-fold serially diluted to different ratios, ranging from 1 to 4 times. The concentration of diluted DNA was measured using a Qubit® fluorometer (Invitrogen, United States). The specificity of the RPA-LFS assay was tested with common plant species that have abundant flavone-related compounds, including *Crataegus pinnatifida*, *Epimedium brevicornu*, *Selaginella tamariscina* and *Arisaema heterophyllum*.

### Data Availability Statement

All necessary information regarding the materials and data are available in the manuscript or in the Supplementary Information.

## Electronic supplementary material

Supplementary Information

## References

[1] Foster BC, Arnason JT, Briggs CJ (2005). Natural Health Products And Drug Disposition*. Annual Review of Pharmacology & Toxicology.

[2] Morris CA, Avorn J (2003). Internet marketing of herbal products. Jama.

[3] Robinson, M. M. & Zhang, X. The World Medicines Situation 2011 Traditional Medicines: Global Situation, Issues And Challenges (2011).

[4] Newmaster SG, Grguric M, Shanmughanandhan D, Ramalingam S, Ragupathy S (2013). DNA barcoding detects contamination and substitution in North American herbal products. Bmc Medicine.

[5] Raclariu, A. C. *et al*. Comparative authentication ofHypericum perforatumherbal products using DNA metabarcoding, TLC and HPLC-MS. *Scientific Reports* **7** (2017). 10.1038/s41598-017-01389-w PMC5431008 28465563

[6] Srirama, R. *et al*. Species Adulteration in the Herbal Trade: Causes, Consequences and Mitigation. *Drug safety*, 1–11 (2017). 10.1007/s40264-017-0527-0 28389979

[7] Diamond BJ (2000). Ginkgo biloba extract: mechanisms and clinical indications. Arch Phys Med Rehabil.

[8] Chen, C. & Luo, S. Studies on the productive technology of Ginkgo Ginkgo biloba extract. *Chinese Traditional & Herbal Drugs* (1997).

[9] Wohlmuth H, Savage K, Dowell A, Mouatt P (2014). Adulteration of Ginkgo biloba products and a simple method to improve its detection. Phytomedicine International Journal of Phytotherapy & Phytopharmacology.

[10] Chandra A (2011). Qualitative categorization of supplement grade Ginkgo biloba leaf extracts for authenticity. Journal of Functional Foods.

[11] Ivanova NV, Kuzmina ML, Braukmann TW, Borisenko AV, Zakharov EV (2016). Authentication of Herbal Supplements Using Next-Generation Sequencing. Plos One.

[12] Ståhlberg, A. *et al*. Simple multiplexed PCR-based barcoding of DNA for ultrasensitive mutation detection by next-generation sequencing. *Nature Protocols* (2017). 10.1038/nprot.2017.006 28253235

[13] Liu, Y., Wang, X., Zitong, G., Han, J. & Xiang, L. Detection of Ophiocordyceps sinensis and its Common Adulterates Using Species-specific Primers (2017). 10.3389/fmicb.2017.01179 PMC5478735 28680424

[14] Wang L, Kong W, Yang M, Han J, Chen S (2015). Safety issues and new rapid detection methods in traditional Chinese medicinal materials. Acta Pharmaceutica Sinica B.

[15] Loo JFC, Lau PM, Ho HP, Kong SK (2013). An aptamer-based bio-barcode assay with isothermal recombinase polymerase amplification for cytochrome- c detection and anti-cancer drug screening. Talanta.

[16] Shen F (2011). Digital Isothermal Quantification of Nucleic Acids via Simultaneous Chemical Initiation of Recombinase Polymerase Amplification Reactions on SlipChip. Analytical Chemistry.

[17] Lutz S (2010). Microfluidic lab-on-a-foil for nucleic acid analysis based on isothermal recombinase polymerase amplification (RPA). Lab on A Chip.

[18] Jing, Z., Dong, H., Dongdong, D. I., Tian, L. & Fan, W. Research Progress on Recombinase Polymerase Amplification (2016).

[19] Yang, Y. *et al*. Development of Isothermal Recombinase Polymerase Amplification Assay for Rapid Detection of Porcine Circovirus Type 2. *Biomed Research International* **2017** (2017). 10.1155/2017/8403642 PMC5382309 28424790

[20] Boyle DS (2013). Rapid detection of HIV-1 proviral DNA for early infant diagnosis using recombinase polymerase amplification. Mbio.

[21] Shalaby MA (2016). Recombinase polymerase amplification assay for rapid detection of lumpy skin disease virus. Journal of Clinical Virology the Official Publication of the Pan American Society for Clinical Virology.

[22] Moore, M. D. & Jaykus, L. A. Development of a Recombinase Polymerase Amplification Assay for Detection of Epidemic Human Noroviruses. *Scientific Reports* **7** (2017). 10.1038/srep40244 PMC5220337 28067278

[23] Yang, L. *et al*. A Nucleotide Signature for the Identification of American Ginseng and Its Products. *Frontiers in Plant Science* **7** (2016). 10.3389/fpls.2016.00319 PMC4796032 27047504

[24] Wang X, Liu Y, Wang L, Han J, Chen S (2016). A Nucleotide Signature for the Identification of Angelicae Sinensis Radix (Danggui) and Its Products. Scientific Reports.

[25] Jia, J., Xu, Z., Xin, T., Shi, L. & Song, J. Quality Control of the Traditional Patent Medicine Yimu Wan Based on SMRT Sequencing and DNA Barcoding. *Frontiers in Plant Science* **8** (2017). 10.3389/fpls.2017.00926 PMC5449480 28620408

[26] Abubakar, B. M., Salleh, F. M., Omar, M. S. S. & Wagiran, A. Review: DNA Barcoding and Chromatography fingerprints For the Authentication of Botanicals in Herbal Medicinal Products: Two Good Heads Are Better than One. *Evidence-Based Complementray and Alternative Medicine,2017,(2017-4-27)* **2017** (2017). 10.1155/2017/1352948 PMC5425840 28536641

[27] Lo YT, Li M, Shaw PC (2015). Identification of constituent herbs in ginseng decoctions by DNA markers. Chinese medicine.

[28] Abbadi, M. *et al*. Species identification of bivalve molluscs by Pyrosequencing™. *Journal of the Science of Food & Agriculture* **97** (2017). 10.1002/jsfa.7754 27068666

[29] Yang Y (2017). Development of real-time and lateral flow strip reverse transcription recombinase polymerase Amplification assays for rapid detection of peste des petits ruminants virus. Virology Journal.

[30] Sharma N, Hoshika S, Hutter D, Bradley KM, Benner SA (2014). Recombinase-based isothermal amplification of nucleic acids with self-avoiding molecular recognition systems (SAMRS). Chembiochem A European Journal of Chemical Biology.

[31] Crannell Z (2016). Multiplexed Recombinase Polymerase Amplification Assay To Detect Intestinal Protozoa. Analytical Chemistry.

