# Supplementary material for: Rapid Authentication of Ginkgo biloba Herbal Products Using the Recombinase Polymerase Amplification Assay
Source: Sci Rep. 2018 May 22;8:8002. doi: 10.1038/s41598-018-26402-8 (PMC5964079; doi:10.1038/s41598-018-26402-8)
Supplement: Supplementary file 1 — Supplementary Information [file 41598_2018_26402_MOESM1_ESM.pdf]

Supplementary Information

Rapid Authentication of Herbal Products by Using Recombinase Polymerase Amplification Assay

Yang Liu, Xiao-yue Wang, Xue-min Wei, Zi-tong Gao, Jian-ping Han *

114-bp *Ginkgo biloba* –specific sequence: CGAGGAAGGTTCTGTTACTAACCTGTTCAC TTCCATTGTAGGGAATGTATTTGGATTCAAAGCCCTACGAGCTCTACGTCTGGAAGATC TGCGAATTCCTCCTGCTTATTCCAA

104-bp *Sophora japonica*-specific sequence: GAGTCTTTGAACGCAAGTTGCGCCCGAAGC CATTAGGCCGAGGGCACGCCTGCCTGGGTGTCACACATCGTTGCCCCAATGCCAGTGC CTCTTGCTAGGTC

Figure S1.

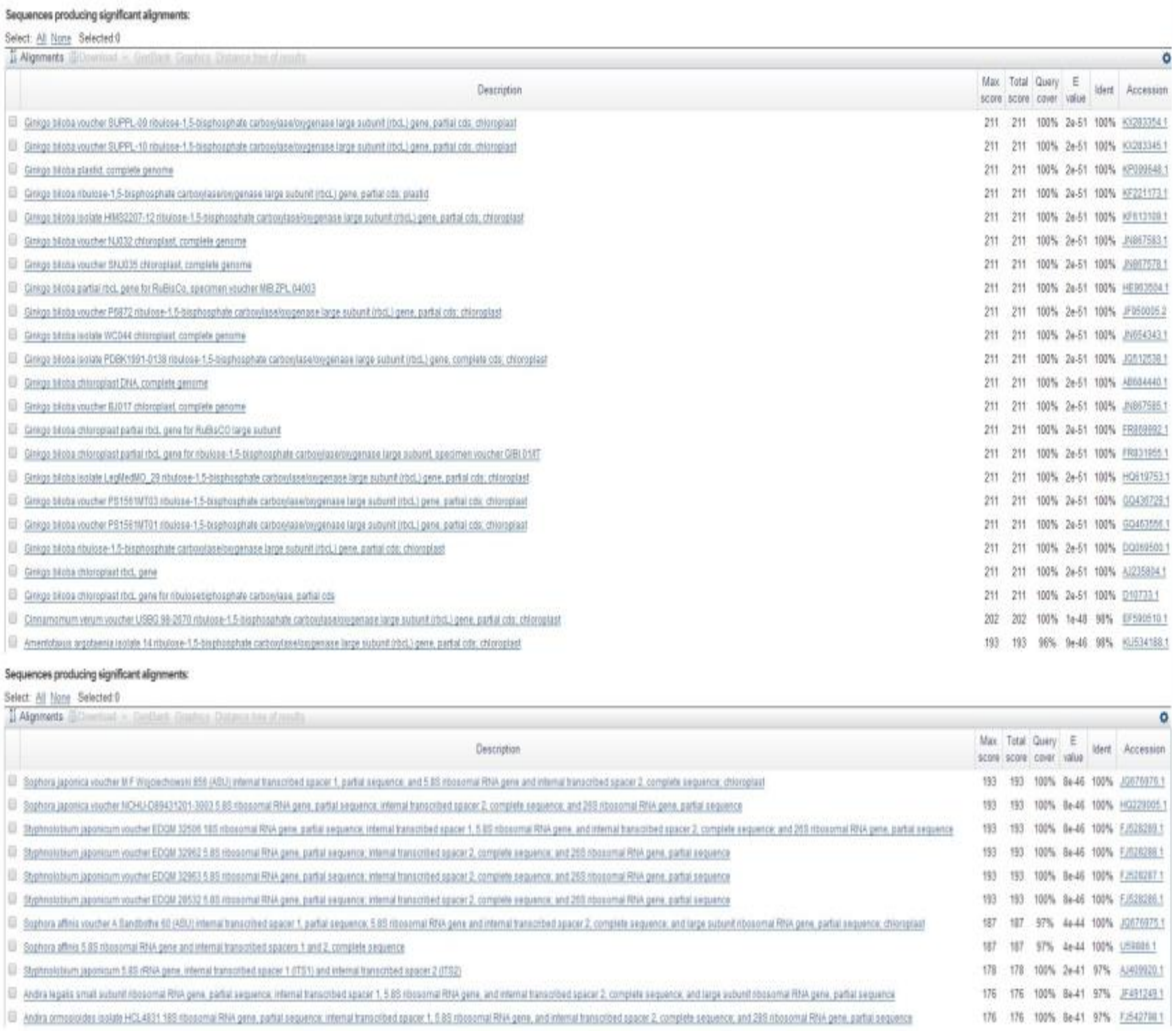

Sequences producing significant alignments:

Select: All None Selected:0

Alignments Download GenBank Graphics Distance tree of results

Description	Max score	Total score	Query cover	E value	Ident	Accession
Ginkgo biloba voucher SUPPL-09 ribulose-1,5-bisphosphate carboxylase/oxygenase large subunit (rbcL) gene, partial cds; chloroplast	211	211	100%	2e-51	100%	KX283354.1
Ginkgo biloba voucher SUPPL-10 ribulose-1,5-bisphosphate carboxylase/oxygenase large subunit (rbcL) gene, partial cds; chloroplast	211	211	100%	2e-51	100%	KX283345.1
Ginkgo biloba plastid, complete genome	211	211	100%	2e-51	100%	KP099648.1
Ginkgo biloba ribulose-1,5-bisphosphate carboxylase/oxygenase large subunit (rbcL) gene, partial cds; plastid	211	211	100%	2e-51	100%	KF221173.1
Ginkgo biloba isolate HMS2207-12 ribulose-1,5-bisphosphate carboxylase/oxygenase large subunit (rbcL) gene, partial cds; chloroplast	211	211	100%	2e-51	100%	KF613109.1
Ginkgo biloba voucher NJ032 chloroplast, complete genome	211	211	100%	2e-51	100%	JN867583.1
Ginkgo biloba voucher SNJ035 chloroplast, complete genome	211	211	100%	2e-51	100%	JN867578.1
Ginkgo biloba partial rbcL gene for RuBisCo, specimen voucher MB.ZPL 04003	211	211	100%	2e-51	100%	HE963504.1
Ginkgo biloba voucher P6872 ribulose-1,5-bisphosphate carboxylase/oxygenase large subunit (rbcL) gene, partial cds; chloroplast	211	211	100%	2e-51	100%	JF950005.2
Ginkgo biloba isolate WC044 chloroplast, complete genome	211	211	100%	2e-51	100%	JN654343.1
Ginkgo biloba isolate PDBK1991-0138 ribulose-1,5-bisphosphate carboxylase/oxygenase large subunit (rbcL) gene, complete cds; chloroplast	211	211	100%	2e-51	100%	JQ512538.1
Ginkgo biloba chloroplast DNA, complete genome	211	211	100%	2e-51	100%	AB684440.1
Ginkgo biloba voucher BJ017 chloroplast, complete genome	211	211	100%	2e-51	100%	JN867585.1
Ginkgo biloba chloroplast partial rbcL gene for RuBisCO large subunit	211	211	100%	2e-51	100%	FR869892.1
Ginkgo biloba chloroplast partial rbcL gene for ribulose-1,5-bisphosphate carboxylase/oxygenase large subunit, specimen voucher GIBI 01IT	211	211	100%	2e-51	100%	FR831955.1
Ginkgo biloba isolate LegMedMO_29 ribulose-1,5-bisphosphate carboxylase/oxygenase large subunit (rbcL) gene, partial cds; chloroplast	211	211	100%	2e-51	100%	HQ619753.1
Ginkgo biloba voucher PS1561MT03 ribulose-1,5-bisphosphate carboxylase/oxygenase large subunit (rbcL) gene, partial cds; chloroplast	211	211	100%	2e-51	100%	GQ436729.1
Ginkgo biloba voucher PS1561MT01 ribulose-1,5-bisphosphate carboxylase/oxygenase large subunit (rbcL) gene, partial cds; chloroplast	211	211	100%	2e-51	100%	GQ463556.1
Ginkgo biloba ribulose-1,5-bisphosphate carboxylase/oxygenase large subunit (rbcL) gene, partial cds; chloroplast	211	211	100%	2e-51	100%	DQ069500.1
Ginkgo biloba chloroplast rbcL gene	211	211	100%	2e-51	100%	AJ235804.1
Ginkgo biloba chloroplast rbcL gene for ribulosebisphosphate carboxylase, partial cds	211	211	100%	2e-51	100%	D10733.1
Cinnamomum verum voucher USBG 98-2670 ribulose-1,5-bisphosphate carboxylase/oxygenase large subunit (rbcL) gene, partial cds; chloroplast	202	202	100%	1e-48	98%	EF590510.1
Amentotaxus argotaenia isolate 14 ribulose-1,5-bisphosphate carboxylase/oxygenase large subunit (rbcL) gene, partial cds; chloroplast	193	193	96%	9e-46	98%	KU534188.1

Sequences producing significant alignments:

Select: All None Selected:0

Alignments Download GenBank Graphics Distance tree of results

Description	Max score	Total score	Query cover	E value	Ident	Accession
Sophora japonica voucher M.F.Wojciechowski 856 (ASU) internal transcribed spacer 1, partial sequence; and 5.8S ribosomal RNA gene and internal transcribed spacer 2, complete sequence; chloroplast	193	193	100%	8e-46	100%	JQ676976.1
Sophora japonica voucher NCHU-Q89431201-3003 5.8S ribosomal RNA gene, partial sequence; internal transcribed spacer 2, complete sequence; and 26S ribosomal RNA gene, partial sequence	193	193	100%	8e-46	100%	HQ229005.1
Styphnolobium japonicum voucher EDQM 32506 18S ribosomal RNA gene, partial sequence; internal transcribed spacer 1, 5.8S ribosomal RNA gene, and internal transcribed spacer 2, complete sequence; and 26S ribosomal RNA gene, partial sequence	193	193	100%	8e-46	100%	FJ528289.1
Styphnolobium japonicum voucher EDQM 32962 5.8S ribosomal RNA gene, partial sequence; internal transcribed spacer 2, complete sequence; and 26S ribosomal RNA gene, partial sequence	193	193	100%	8e-46	100%	FJ528288.1
Styphnolobium japonicum voucher EDQM 32963 5.8S ribosomal RNA gene, partial sequence; internal transcribed spacer 2, complete sequence; and 26S ribosomal RNA gene, partial sequence	193	193	100%	8e-46	100%	FJ528287.1
Styphnolobium japonicum voucher EDQM 28532 5.8S ribosomal RNA gene, partial sequence; internal transcribed spacer 2, complete sequence; and 26S ribosomal RNA gene, partial sequence	193	193	100%	8e-46	100%	FJ528286.1
Sophora affinis voucher A.Sandbothe 60 (ASU) internal transcribed spacer 1, partial sequence; 5.8S ribosomal RNA gene and internal transcribed spacer 2, complete sequence; and large subunit ribosomal RNA gene, partial sequence; chloroplast	187	187	97%	4e-44	100%	JQ676975.1
Sophora affinis 5.8S ribosomal RNA gene and internal transcribed spacers 1 and 2, complete sequence	187	187	97%	4e-44	100%	U59886.1
Styphnolobium japonicum 5.8S rRNA gene, internal transcribed spacer 1 (ITS1) and internal transcribed spacer 2 (ITS2)	178	178	100%	2e-41	97%	AJ409920.1
Andira legalis small subunit ribosomal RNA gene, partial sequence; internal transcribed spacer 1, 5.8S ribosomal RNA gene, and internal transcribed spacer 2, complete sequence; and large subunit ribosomal RNA gene, partial sequence	176	176	100%	8e-41	97%	JF481249.1
Andira ormosioides isolate HCL4831 18S ribosomal RNA gene, partial sequence; internal transcribed spacer 1, 5.8S ribosomal RNA gene, and internal transcribed spacer 2, complete sequence; and 28S ribosomal RNA gene, partial sequence	176	176	100%	8e-41	97%	FJ542798.1

Figure S1. BLAST results of species-specific sequences

Figure S2. BLAST results of species-specific probes

Sequences producing significant alignments:

Select: All None Selected:0

Alignments Download GenBank Graphics Distance tree of results

Description	Max score	Total score	Query cover	E value	Ident	Accession
Ginkgo biloba voucher SUPPL-09 ribulose-1,5-bisphosphate carboxylase/oxygenase large subunit (rbcL) gene, partial cds; chloroplast	89.8	89.8	100%	3e-15	100%	KX283354.1
Ginkgo biloba voucher SUPPL-10 ribulose-1,5-bisphosphate carboxylase/oxygenase large subunit (rbcL) gene, partial cds; chloroplast	89.8	89.8	100%	3e-15	100%	KX283345.1
Ginkgo biloba voucher BOP010383 ribulose-1,5-bisphosphate carboxylase/oxygenase large subunit (rbcL) gene, partial cds; chloroplast	89.8	89.8	100%	3e-15	100%	KP088621.1
Ginkgo biloba plastid, complete genome	89.8	89.8	100%	3e-15	100%	KF099648.1
Ginkgo biloba ribulose-1,5-bisphosphate carboxylase/oxygenase large subunit (rbcL) gene, partial cds; plastid	89.8	89.8	100%	3e-15	100%	KF221173.1
Ginkgo biloba isolate HMS2207-12 ribulose-1,5-bisphosphate carboxylase/oxygenase large subunit (rbcL) gene, partial cds; chloroplast	89.8	89.8	100%	3e-15	100%	KF813108.1
Ginkgo biloba voucher NJ032 chloroplast, complete genome	89.8	89.8	100%	3e-15	100%	JN867583.1
Ginkgo biloba voucher SNJ035 chloroplast, complete genome	89.8	89.8	100%	3e-15	100%	JN867578.1
Ginkgo biloba partial rbcL gene for RuBisCo, specimen voucher MB ZPL 04003	89.8	89.8	100%	3e-15	100%	HE963504.1
Ginkgo biloba voucher P6872 ribulose-1,5-bisphosphate carboxylase/oxygenase large subunit (rbcL) gene, partial cds; chloroplast	89.8	89.8	100%	3e-15	100%	JF950005.2
Ginkgo biloba isolate PDBK1991-0138 ribulose-1,5-bisphosphate carboxylase/oxygenase large subunit (rbcL) gene, complete cds; chloroplast	89.8	89.8	100%	3e-15	100%	JQ512538.1
Ginkgo biloba chloroplast DNA, complete genome	89.8	89.8	100%	3e-15	100%	AB684440.1
Ginkgo biloba voucher BJ017 chloroplast, complete genome	89.8	89.8	100%	3e-15	100%	JN867585.1
Ginkgo biloba chloroplast partial rbcL gene for RuBisCO large subunit	89.8	89.8	100%	3e-15	100%	FR869892.1
Ginkgo biloba chloroplast partial rbcL gene for ribulose-1,5-bisphosphate carboxylase/oxygenase large subunit, specimen voucher GIBI 01MT	89.8	89.8	100%	3e-15	100%	FR831955.1
Ginkgo biloba isolate LegMedMO_29 ribulose-1,5-bisphosphate carboxylase/oxygenase large subunit (rbcL) gene, partial cds; chloroplast	89.8	89.8	100%	3e-15	100%	HQ619753.1
Ginkgo biloba voucher PS1561MT03 ribulose-1,5-bisphosphate carboxylase/oxygenase large subunit (rbcL) gene, partial cds; chloroplast	89.8	89.8	100%	3e-15	100%	GQ436729.1
Ginkgo biloba voucher PS1561MT01 ribulose-1,5-bisphosphate carboxylase/oxygenase large subunit (rbcL) gene, partial cds; chloroplast	89.8	89.8	100%	3e-15	100%	GQ463556.1
Ginkgo biloba ribulose-1,5-bisphosphate carboxylase/oxygenase large subunit (rbcL) gene, partial cds; chloroplast	89.8	89.8	100%	3e-15	100%	DQ069500.1
Ginkgo biloba chloroplast rbcL gene	89.8	89.8	100%	3e-15	100%	AJ235804.1
Ginkgo biloba chloroplast rbcL gene for ribulosebiphosphate carboxylase, partial cds	89.8	89.8	100%	3e-15	100%	D10733.1

Sequences producing significant alignments:

Select: All None Selected:0

Alignments Download GenBank Graphics Distance tree of results

Description	Max score	Total score	Query cover	E value	Ident	Accession
Sophora japonica isolate PB032MT2 internal transcribed spacer 2, partial sequence	87.9	87.9	100%	9e-15	100%	KT285088.1
Sophora japonica isolate PB032MT3 internal transcribed spacer 2, partial sequence	87.9	87.9	100%	9e-15	100%	KT285087.1
Sophora japonica isolate PB032MT1 internal transcribed spacer 2, partial sequence	87.9	87.9	100%	9e-15	100%	KT285086.1
Sophora japonica voucher YC0180MT14 internal transcribed spacer 2, partial sequence	87.9	87.9	100%	9e-15	100%	KT898231.1
Sophora japonica voucher NCHU-D89431201-3003 5.8S ribosomal RNA gene, partial sequence; internal transcribed spacer 2, complete sequence; and 26S ribosomal RNA gene, partial sequence	87.9	87.9	100%	9e-15	100%	HQ229005.1
Styphnolobium japonicum voucher EDQM 32506 18S ribosomal RNA gene, partial sequence; internal transcribed spacer 1, 5.8S ribosomal RNA gene, and internal transcribed spacer 2, complete sequence; and 26S ribosomal RNA gene, partial sequence	87.9	87.9	100%	9e-15	100%	FJ528289.1
Styphnolobium japonicum voucher EDQM 32962 5.8S ribosomal RNA gene, partial sequence; internal transcribed spacer 2, complete sequence; and 26S ribosomal RNA gene, partial sequence	87.9	87.9	100%	9e-15	100%	FJ528288.1
Styphnolobium japonicum voucher EDQM 32963 5.8S ribosomal RNA gene, partial sequence; internal transcribed spacer 2, complete sequence; and 26S ribosomal RNA gene, partial sequence	87.9	87.9	100%	9e-15	100%	FJ528287.1
Styphnolobium japonicum voucher EDQM 28532 5.8S ribosomal RNA gene, partial sequence; internal transcribed spacer 2, complete sequence; and 26S ribosomal RNA gene, partial sequence	87.9	87.9	100%	9e-15	100%	FJ528286.1
Sophora japonica voucher M.F.Wojciechowski 856 (ASU) internal transcribed spacer 1, partial sequence; and 5.8S ribosomal RNA gene and internal transcribed spacer 2, complete sequence; chloroplast	82.4	82.4	100%	4e-13	98%	JQ676976.1
Sophora japonica ITS2	82.4	82.4	100%	4e-13	98%	Z72341.1
Sophora affinis voucher A.Sandbothe 60 (ASU) internal transcribed spacer 1, partial sequence; 5.8S ribosomal RNA gene and internal transcribed spacer 2, complete sequence; and large subunit ribosomal RNA gene, partial sequence; chloroplast	65.8	65.8	74%	4e-08	100%	JQ676975.1
Sophora affinis 5.8S ribosomal RNA gene and internal transcribed spacers 1 and 2, complete sequence	65.8	65.8	74%	4e-08	100%	U59886.1

Figure S2. BLAST results of species-specific probes

Figure S3.

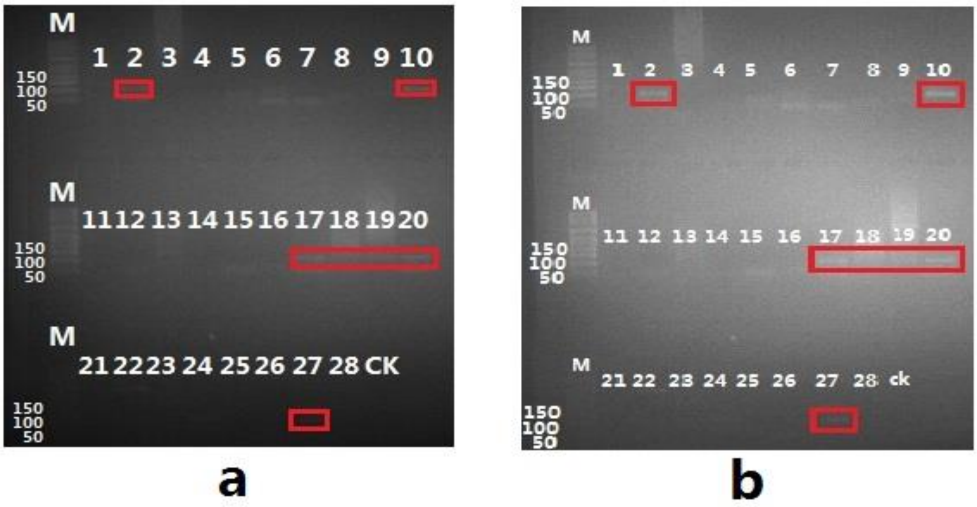

Figure S3. PCR Amplification Results of NHP with Primer Pair HMF/HMR under Multiple Exposures. S3a is the normally exposed gel image. S3b is the overexposed gel image.

TABLE S1. Information for the samples of *Ginkgo biloba* tea and leave extract used in this study

Sample No.	Sample Type	Production Place	Sequences Analysis Result	
			Ginkgo biloba	*Sophora japonica*
T01	Tea	Hubei, China	+	-
T02	Tea	Zhejiang, China	+	-
T03	Tea	Shandong, China	+	-
T04	Tea	Shandong, China	+	-
T05	Tea	Hebei, China	+	-
T06	Tea	Hubei ,China	+	-
T07	Tea	Hebei, China	+	-
T08	Tea	Unknown China	+	-
NHP01	Capsule	Shandong, China	+	-
NHP02	Powder	Shaanxi, China	+	+
NHP03	Capsule	Dusseldorf, Germany	+	-
NHP04	Capsule	Fargo, America	+	-
NHP05	Powder	Sichuan, China	+	-
NHP06	Powder	Sichuan, China	+	-
NHP07	Powder	Sichuan, China	+	-
NHP08	Capsule	Shandong, China	+	-
NHP09	Powder	Shaanxi, China	-	-
NHP10	Powder	Shaanxi, China	+	+
NHP11	Tablet	Collingwood, Australia	-	-
NHP12	Capsule	Zhejiang, China	+	-
NHP13	Powder	Jiangsu, China	+	-
NHP14	Tablet	Yokohama, Japan	+	-
NHP15	Capsule	Illinois, America	+	-
NHP16	Powder	Shaanxi, China	+	-
NHP17	Tablet	Tokyo, Japan	+	+
NHP18	Powder	Beijing, China	+	+
NHP19	Powder	Shaanxi, China	+	+
NHP20	Powder	Jiangsu, China	+	+
NHP21	Capsule	San clemente, America	+	-
NHP22	Powder	Henan, China	+	-
NHP23	Capsule	Guangdong, Chian	+	-
NHP24	Capsule	New York, America	+	-
NHP25	Capsule	California,	+	-

		America		
NHP26	Tablet	Hiroshima, Japan	+	-
NHP27	Powder	Shaanxi, China	+	+
NHP28	Capsule	Flensburg, Germany	+	-
